# P-103. Landscape of Post-Marketing Requirements under the Pediatric Research Equity Act for Antibiotics from 2009-2024

**DOI:** 10.1093/ofid/ofaf695.332

**Published:** 2026-01-11

**Authors:** Daniel Selig, Funmi Aminu, Sue K Cammarata, Ting Chen, Lauren Dolak, Stephen Duprez, Stephanie Ecker, Lisa Gault, Sandra George, Margaret Harkins, Clayton Litchmore, Michael Serenko, William Waverczak, Douglas Girgenti

**Affiliations:** Melinta Therapeutics, Parsippany, New Jersey; Melinta Therapeutics, Parsippany, New Jersey; Tunnell, Chicago, Illinois; Melinta Therapeutics, Parsippany, New Jersey; Melinta Therapeutics, Parsippany, New Jersey; Melinta Therapeutics, Parsippany, New Jersey; Melinta Therapeutics, Parsippany, New Jersey; Melinta Therapeutics, Parsippany, New Jersey; Melinta Therapeutics, Parsippany, New Jersey; Melinta Therapeutics, Parsippany, New Jersey; Melinta Therapeutics, Parsippany, New Jersey; Melinta Therapeutics, Parsippany, New Jersey; Melinta Therapeutics, Parsippany, New Jersey; Melinta Therapeutics, Parsippany, New Jersey

## Abstract

**Background:**

We reviewed Post-Marketing Requirements (PMRs) under the Pediatric Research Equity Act (PREA) for antibiotics approved in adults from 2009 to 2024 indicated for acute bacterial skin and structural infections (ABSSSI), community-, hospital-acquired-, ventilator-associated bacterial pneumonia (CABP, HABP, and VABP), complicated urinary tract infection (cUTI) or complicated intra-abdominal infection (cIAI), to better understand PMR completion.

**Figure:**
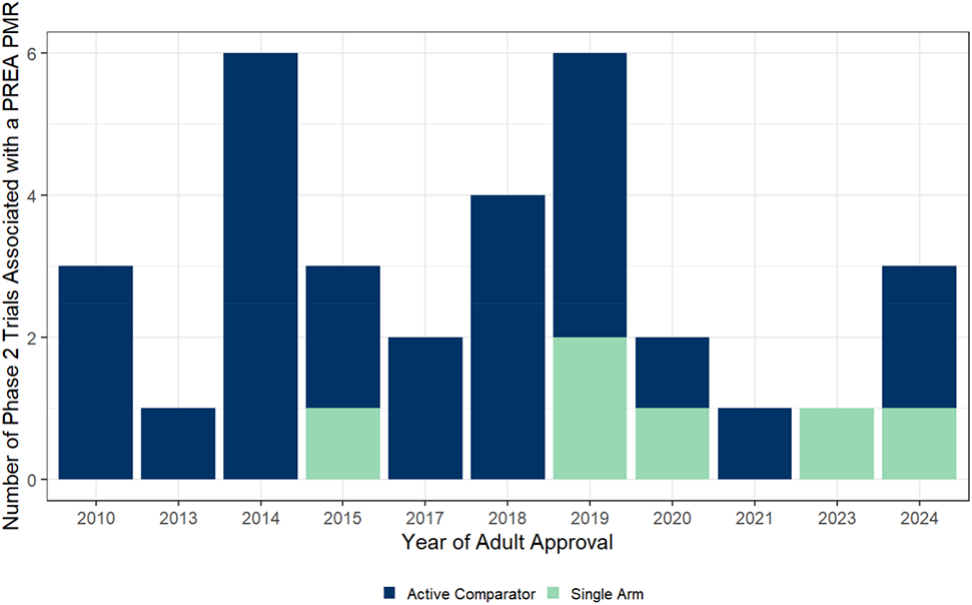
Trends in Utilization of Single Arm Study Design for Pediatric Antibiotic Trials under PREA

A summary of the number of Phase 2 studies associated with a PREA PMR that had an active comparator or single-arm design. These study designs were the initial proposed designs agreed upon by the Sponsor and the FDA. Note that Sponsors may update study designs in consultation with the FDA, so this may underestimate the number of single arm phase 2 pediatric antibiotic trials currently utilized to fulfill PREA PMR requirements.

**Figure:**
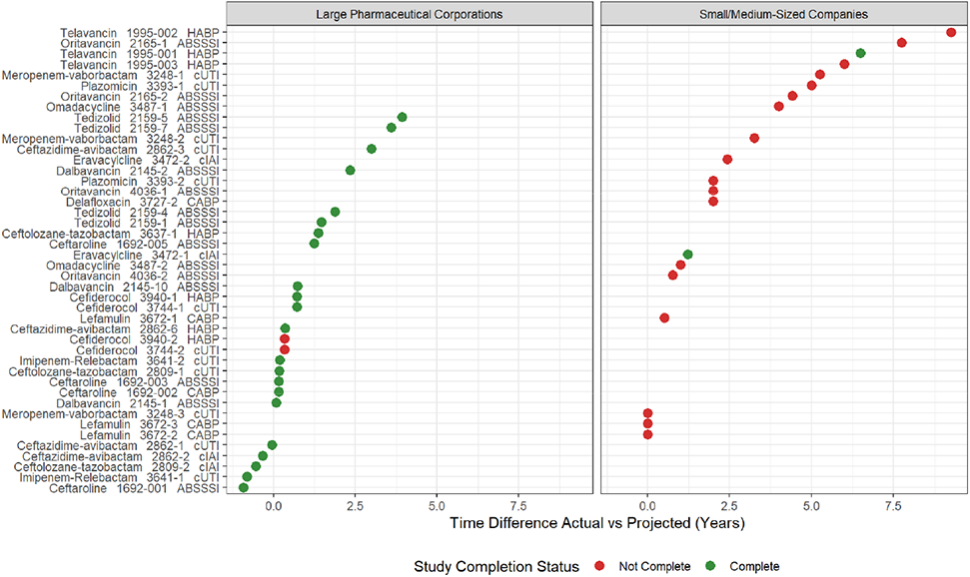
Completion Status of PMR Associated Studies for Large and Small/Medium Sized Pharmaceutical Companies

Summary of PREA PMR-associated studies that have completed, PREA PMR-associated studies that have not yet completed, and the difference between projected and actual time to complete a study associated with a PREA PMR. The projected time for study completion was the initial agreement between the Sponsor and the FDA as cited in the respective adult approval letters. Circles represent the difference in actual vs. projected study completion time for studies associated with a PREA PMR (PMR number provided in y-label) Time differences that are less than 0 represent studies that were completed prior to the projected date

**Methods:**

Initial PMRs, including study design and completion timelines were extracted from Food and Drug Administration (FDA) approval letters. Studies were cross-referenced at clinicaltrials.gov, with follow-up from adult approval to study completion or through 01/01/2025. Cumulative Incidence of Study Completion From Initial Adult FDA Approval

**Figure:**
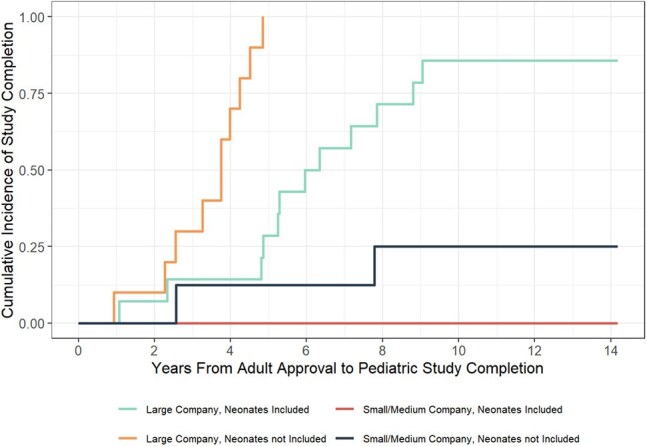
Cumulative incidence of pediatric study completion from adult approval date by company size and inclusion of a neonatal population

**Results:**

Eighteen antibiotics were approved in adults from 2009 to 2024, with 53 associated PREA PMRs. Of 44 PMRs in the analysis set, median pediatric study follow-up time from adult approval was 5.3 years (range 0.94 to 11.5 years), with study completion rate of 54.5% (N = 24). Small/medium-sized companies had a study completion rate of 10% (N = 2/20) over a median 6.44 years of follow-up, with no pediatric approvals. Large pharmaceutical corporations had a significantly higher study completion rate of 91.6% (N = 22/24, adjusted hazard ratio 20.3 95%CI, 5.02 to 82.4) over a median follow-up time of 4.7 years, and achieved pediatric approval with labelling updates for 75% of antibiotics (N = 6/8).

**Conclusion:**

Compared to larger organizations, smaller pharmaceutical companies have experienced difficulty in PREA PMR antibiotic study completion, which may be related to financial difficulties in the challenging market for antibiotics. To improve PMR study completion, smaller companies require continued financial support and innovation in study design. For pediatric antibiotic development, the FDA accepts extrapolation of efficacy from well-conducted randomized adult trials (i.e. pharmacokinetics (PK) and safety approach). Sponsors should consider using single-arm, non-comparative PK and safety study designs to reduce trial size and scope. Sponsors should also assess whether evaluation of an antibiotic is necessary in adolescents, or if data in a surrogate population of adults (e.g., low-weight adults) may serve as adequate evidence for approval.

**Disclosures:**

Daniel Selig, MD, Melinta Therapeutics: Employee|Melinta Therapeutics: Stocks/Bonds (Private Company) Funmi Aminu, MD, Melinta Therapeutics: Employee|Melinta Therapeutics: Stocks/Bonds (Private Company)|Melinta Therapeutics: Stocks/Bonds (Private Company) Ting Chen, MS, Melinta Therapeutics: Stocks/Bonds (Private Company) Lauren Dolak, MS, Melinta Therapeutics: Employee Stephen Duprez, MS, Melinta Therapeutics: Employee of Melinta Therapeutics and received “stock” as part of a private company Stephanie Ecker, MS, Melinta Therapeutics: Employee Lisa Gault, BA, Melinta Therapeutics: Employee Sandra George, BA, Melinta Therapeutics: Employee Margaret Harkins, RN, n/a, Melinta Therapeutics LLC: Stocks/Bonds (Private Company) Clayton Litchmore, MS, RAC, Melinta Therapeutics, LLC: Stocks/Bonds (Private Company) Michael Serenko, MD, Melinta: Stocks/Bonds (Private Company) Douglas Girgenti, MD, Melinta: Employee|Melinta: Stocks/Bonds (Private Company)

